# Let-Us Investigate; A Meta-Analysis of Influencing Factors on Lettuce Crop Yields within Controlled-Environment Agriculture Systems

**DOI:** 10.3390/plants12142623

**Published:** 2023-07-12

**Authors:** Michael Gargaro, Richard J. Murphy, Zoe M. Harris

**Affiliations:** Centre for Environment and Sustainability, University of Surrey, Guildford GU2 7XH, UK; m.gargaro@surrey.ac.uk (M.G.); rj.murphy@surrey.ac.uk (R.J.M.)

**Keywords:** lettuce, yield, controlled-environment agriculture, vertical farming, *Lactuca sativa*, meta-analysis, production, hydroponics, fresh weight

## Abstract

Climate change-related impacts have hampered the productivity of agricultural lands in recent times, affecting food security globally. Novel technology-based agricultural production systems such as controlled-environment agriculture (CEA) are a way to reduce the impact of climatic variation and pests that harm current global crop production and ensure consistent crop development. These systems often use artificial lighting and soilless mediums to produce crops. This meta-analysis has investigated the key influencing factors on crop production within these systems, using previous studies on lettuce (the most cultivated crop in these systems) to understand what affects yield within CEA. This analysis has found that on average, CEA systems yield twice that of field-based agriculture (3.68 kg m^−2^ vs. 1.88 kg m^−2^), with the most influencing factors being the variety of cultivars grown, the season, the nutrient delivery method, and the lighting type. The cultivation time for this study was 40 days, with 94% of papers having trial periods of 70 days or less, much lower than field-based agriculture (60–120 days). Vertical farming (stacked vertical CEA cultivation) studies were found to especially drive up yield per area (6.88 kg m^−2^). The results of this meta-analysis are useful for starting to understand the key influencing factors on CEA growth and highlight the breadth of research ongoing in the CEA industry.

## 1. Introduction

In recent years, climate change-related impacts on agricultural production and food security have become a serious challenge. Drought and land degradation caused by climate change and land-use intensification have prompted innovation throughout the sector to grow more food on land that is becoming increasingly difficult to cultivate [1,2]. With the global population predicted to increase by two billion (bn) people in the next 30 years, from 7.7 bn to 9.7 bn by 2050 and potentially peaking at nearly 11 bn by 2100, there is a clear incentive to find alternative, complimentary cultivation systems to cope with rapid population growth and globally diminishing arable land area [3]. Alongside population growth, migration to urban centres is predicted to increase even further, with around 2.5 bn more people living in urban settings than at present [4]. With a rapidly growing urban population and waning agricultural land, exploring novel food production systems will be key to ensuring global prosperity and food security moving forward.

Controlled-Environment Agriculture (CEA) is a technology-based crop production system comprised of various nutrient delivery systems (including hydroponics, aeroponics and aquaponics), which optimise the use of resources such as water, energy and space, producing crops all year round [5]. Within CEA, crops are farmed with optimised growth conditions, allowing greater control over cultivation [6]. At present, CEA offers many benefits by avoiding the impact of external factors and ensuring a protected environment for plant growth. New innovative farming methods such as vertical farming (VF) come under the umbrella of CEA, providing even more benefits by using the vertical plane to maximise land use.

When growing plants in an open field, yield and quality are dependent on weather conditions and other externalities, whereas CEA is designed to avoid these impacts [6]. If planned and managed properly, there are many potential advantages over conventional production systems. CEA systems can be built anywhere; the closed system growing environment is not affected by the outside climate and soil fertility, as well as being productive all year round, pesticide-free, and with high resource use efficiency compared to field-based agriculture [7]. It is worth noting that these systems can have an environmental impact, notably through the energy required for artificial lighting and the maintenance of a controlled environment (heating, cooling, etc.) [8]. With current electrical energy from the grid still largely composed of fossil sources, the energy demand of CEA systems can thus become a major environmental concern and contributor to global climate change. Despite the introduction of new developments and innovations to increase energy efficiency in CEA, systems will remain energy-intensive, and the need for cleaner energy sources is gaining importance [8].

Currently, economic constraints limit crop suitability within CEA systems (in particular within VF systems), meaning only smaller crops (30 cm or shorter in height) such as leafy greens are economically feasible within these systems [7]. Compared to field-based agriculture, CEA has high capital costs (CEA construction, specialised growing racks, etc.) as well as operational costs (controlling the environment, highly skilled labour, etc.), making VF (and CEA more generally) currently suited for quick-growing crops that can retail at premium prices. Lettuce (*Lactuca sativa*) is a high-value food commodity, being one of the most economically important leafy vegetables in the world, worth $2.93 bn USD annually with a Compound Annual Growth Rate (CAGR) of 2.52% [9,10]. The high price and returns from lettuce, as well as its rapid growth rate, make it an ideal plant within CEA. At present, lettuce prices from vertical farms are averaging $7.82 USD per kg of lettuce, over double the business-as-usual field production of $3.04 USD [11]. 

Despite a number of studies on lettuce growth within CEA, to date, there has been no synthesis of these studies results. At present, there is no consolidated growth rate value that industry and researchers alike can use to influence decision-making. This study will assess the state of the literature on lettuce yields within CEA systems. A systematic search will encapsulate all relevant papers, followed by a meta-analysis to evaluate factors within growth trials that influence CEA lettuce cultivation. It will provide a useful conclusive value on yields within previous studies, present a more precise estimate of the effect size, and increase the reliability of the results of individual studies [12]. 

## 2. Results

### 2.1. Systematic Search Results

**Figure 1:**
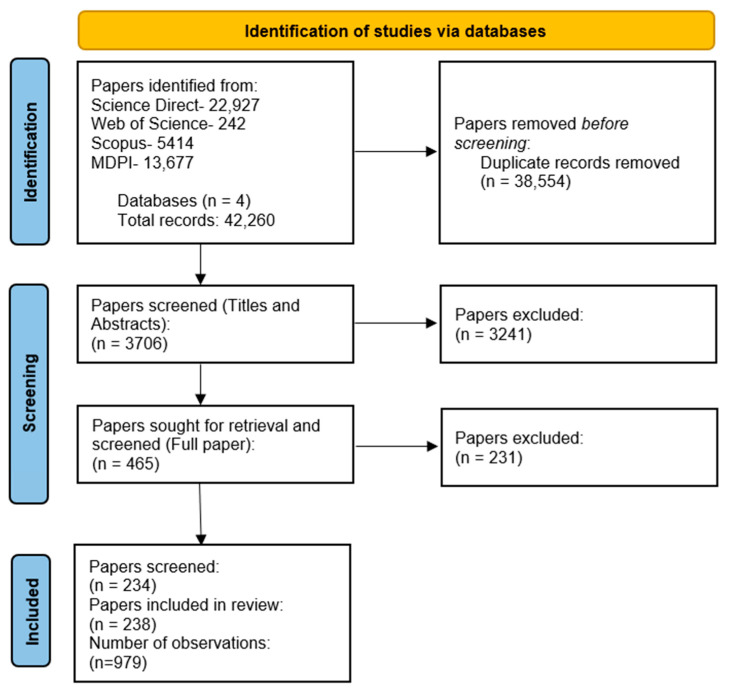
PRISMA flow diagram of the systematic search process completed as part of this study. It highlights the steps taken in identifying studies to be included in the analysis, including the number of papers included or excluded at any stage. Overall, 121 papers were included in the search, totaling 979 observations.

The literature review search returned 3706 publications (after the removal of duplicates), yielding 121 papers that satisfied inclusion criteria, totaling 979 total observations. Many more publications were acknowledged as being appropriate for inclusion, but missing crucial data resulted in many papers being excluded. Figure 1 outlines the systematic search process, highlighting the number of papers identified, screened, and finally included. Table 1 highlights the data parameters extracted from every study that were used as variables in the random and mixed effects models run in this analysis. These categorical and continuous variables were extracted and used for subgroup analysis and meta-regression to further explain and give context to the results and insight into which factors influence lettuce yields.

### 2.2. Description of the Dataset

**Figure 2:**
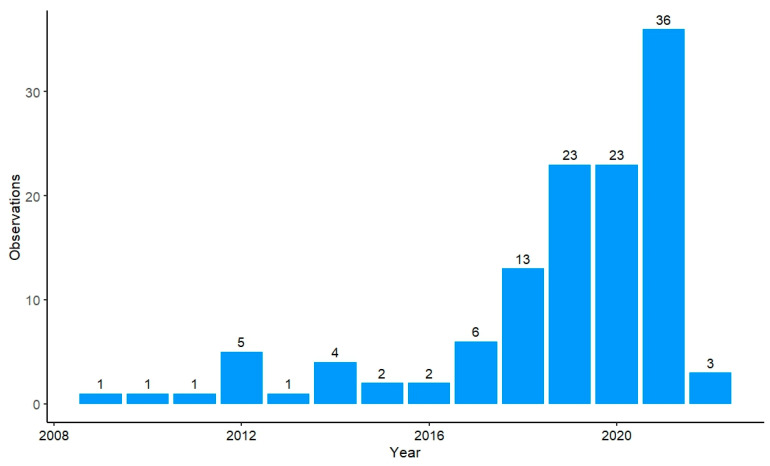
Total number of publications per year between 2009 and 2022.

The number of papers (different from the number of observations, represented by n) that present the yield of lettuce crops in controlled growth trials has increased between 2009 and 2022. There has been an exponential increase in the number of publications, especially from 2017 to 2021, with this time period accounting for 85% of all publications. All years prior to this, as seen in Figure 2, ranged from one to six papers per year that satisfied this meta-analysis’ inclusion criteria. With respect to varieties, ‘looseleaf’ (n = 266) followed by ‘butterhead’ (n = 221) were the most common lettuce varieties used in these trials, though varieties such as ‘batavia’ and ‘cos’ were also commonly cultivated (n = 152 and n = 129, respectively).

**Figure 3:**
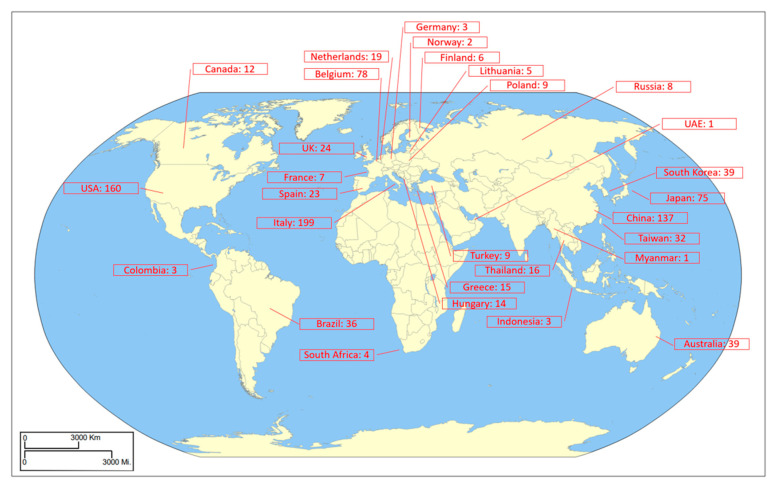
Global map indicating the total number of observations per country.

The treatment interventions (the experimental categories) used within the studies evaluated were broad. Manipulating nutrient solutions (n = 429) and lighting setups (n = 371) were the most popular from the 979 total observations. For the lighting and nutrient categories, factors were noted down, regardless of whether they were increasing or decreasing exposure/time-duration or composition/concentration of the tested treatment. Due to this, results from the global analysis need to be interpreted carefully, especially as this meta-analysis encompasses a wide diversity of systems. Nutrient manipulation included testing aquaponic nutrient solutions, the impact of novel nutrient solutions, variable conductivity and pH, the type of delivery system, and intervals between nutrient delivery, all relevant but vastly different in how the trials were designed and performed. Lighting manipulation was similar in variability, with photoperiod, light intensity, shading, and lighting type all being tested; again, all suitable for inclusion in this meta-analysis but with varying results. Other factors were also evaluated throughout the literature, ranging from seasonal impacts to the cultivation medium used (Full list available in Appendix SA). This gave quite a wide range of treatment interventions and revealed the real breadth of research conducted within CEA systems on manipulating lettuce crop growth. Trials were conducted in a wide variety of nations as well (Figure 3), with Italy having the most observations (n = 199), followed by the USA (n = 160) and China (n = 137). 

### 2.3. Lettuce Yield Results: Global Analysis Results

For the overall effect size calculation, heterogeneity was assessed using the inconsistency index (I^2^) and Cochrane’s Q. The Q statistic indicated that all studies showed a significant degree of between-study heterogeneity, suggesting that heterogeneity is due to statistical, not systematic, uncertainty. The I^2^ index is defined as the percentage of variability in effect sizes that is not caused by sampling error. In this study, the I^2^ statistic equaled 100%, indicating that all variability was due to between-study heterogeneity. 

**Figure 4:**
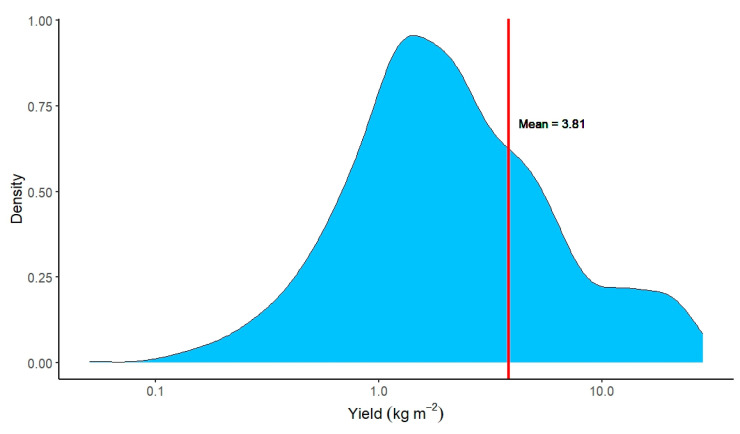
Distribution of true effect sizes (yield), including the mean value (red line) from the global analysis, on a logarithmic scale.

Figure 4 shows the distribution of true effect sizes with the inclusion of the mean value from our global analysis, which was 3.68 kg m^−2^ 95%CI (3.38–3.98). From Figure 4, we can see that the data is asymmetrical and right-skewed, indicating there are many papers with smaller values. 

Due to the high between-study heterogeneity observed in this study (as a consequence of the diverse variety of systems assessed), the value of the global result must be evaluated with caution, hence the need for subgroup analysis and meta-regression. The *p*-value for the global result was equal to zero, indicating that the results are statistically significant even if there is a high amount of variance and between-study heterogeneity. Heterogeneity plots are available in Appendix SB. 

### 2.4. Subgroup Analysis and Meta-Regression Results

#### 2.4.1. Vertically Grown Crops (n = 144)

To account for the difference that stacking in vertical cultivation can cause, a subgroup analysis of systems that only grew crops vertically was performed in order to understand the potential benefits of these systems. From this subgroup analysis (where a new meta-analysis with only studies that had vertically grown lettuce was performed), the average yield of crops grown in vertical systems was 6.88 kg m^−2^, higher than the global analysis result of 3.68 kg m^−2^, and the FAO field results of 1.88 kg m^−2^. Vertically stacked growth is an important avenue for controlled-environment agriculture overall, and this higher yield per area will be discussed.

#### 2.4.2. Impact of Time (n = 957)

**Figure 5:**
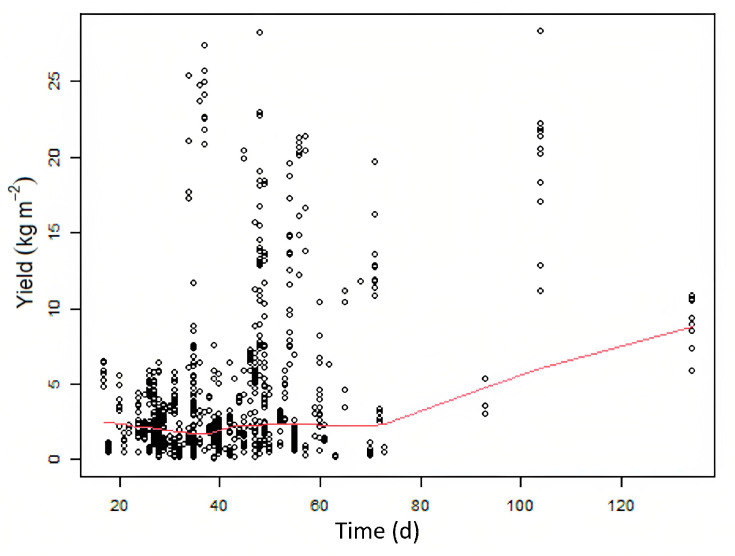
Regression model between time and yield. Each dot represents a different study (effect size), the red line shows the regression line.

Meta-regression results present the amount of heterogeneity that time accounts for (R^2^), given as a percentage of the total heterogeneity found in this study. Time, referring to the amount of time to harvestable size, accounts for ~15% of heterogeneity in this study, which is quite a high proportion. When testing for multi-collinearity, we evaluated the relationship between time and yield, with results indicating a mild positive correlation (0.38), unsurprisingly confirming that the longer a lettuce crop is growing, the higher the yield (Figure 5). The average time to harvestable size for this study was 40 days, with 94% of papers having trial periods of 70 days or less. The time needed is considerably lower than field-based cultivation (60 to 120 days) [13]. 

#### 2.4.3. Impact of Different Lettuce Varieties (n = 979)

**Figure 6:**
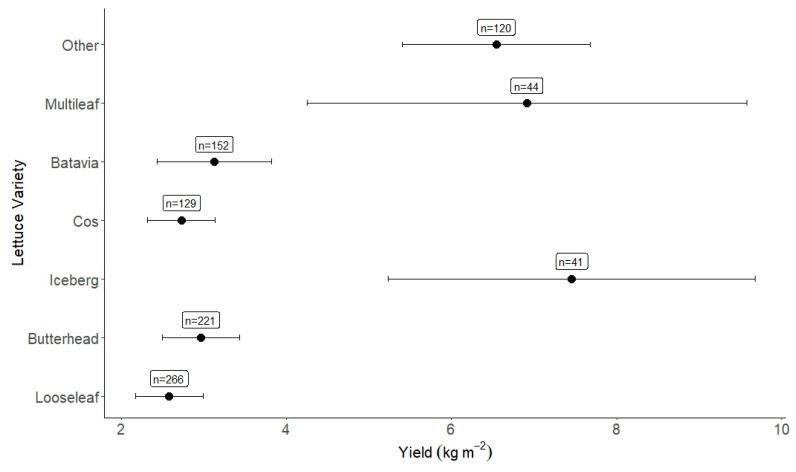
Yield per lettuce variety. The number of observations is given alongside the effect size result.

Different varieties of the same plant will all vary in their size, taste and appearance, and hence also differ in their growth characteristics. In our analysis, we grouped together lettuce by its variety groups: ‘butterhead’, ‘batavia’, ‘iceberg’, ‘looseleaf’, ‘multileaf’ and ‘cos’, as well as an ‘other’ category for lettuce cultivars that were not labelled as being part of the above classifications. Generally, papers identified the lettuce varieties grown as either the variety grouping or the specific cultivar name (i.e., *Lactuca sativa* var. *crispa* is a type of ‘looseleaf’ lettuce). Subgroup analysis was completed on variety type (Figure 6) and found ‘iceberg’ lettuce as the highest yielding (7.45 kg m^−2^, n = 41), much higher than the lowest yielding cultivar, which was ‘looseleaf’ (2.58 kg m^−2^, n = 266). From completing meta-regression on cultivar against yield, the cultivar chosen had a ~12% influence on the heterogeneity displayed in the results; this is significant but demonstrates there are differences between cultivar types and their impact on yield. This study found that icebergs have the highest yield but also, on average, take longer to reach harvestable levels (41 days) than looseleaf (37 days). 

This meta-analysis is measuring the yield of lettuce, and as variety is an independent variable, it must be evaluated with caution. Though variety is a factor, knowing that different cultivars can reach different sizes means that we must account for this fact; we are evaluating the influencing factors of lettuce, not using a variety as a factor that drives different yields. The impact of variety has the potential to create bias, and controlling its influence by sub-grouping all data by cultivar and running the meta-analysis has allowed the impact of variety to be accounted for. Results from the individual meta-analyses of varieties have revealed that the cultivar does not impact the reliability of the results, with each cultivar following a very similar pattern of results when subgrouped by the various independent variables chosen (Appendix SC). There are differences in actual yield values between varieties, but this is due to other factors that have been accounted for in this study and the different characteristics of each variety, not the variety itself.

#### 2.4.4. Impact of Building Type (n = 979) and Season (n = 417)

**Figure 7:**
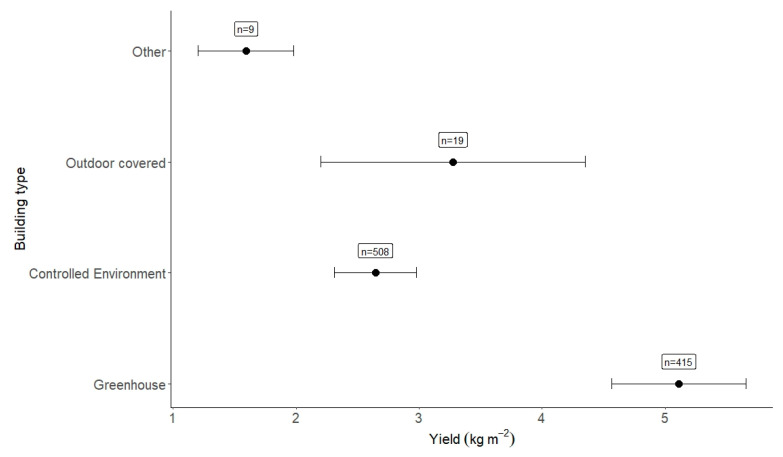
Yield per building type. The number of observations is given alongside the effect size result.

The building type in this study accounted for ~6.5% of the heterogeneity observed. The highest-yielding building type in this experiment was greenhouses (5.11 kg m^−2^, n = 415). This result was followed by outdoor covered areas (which include polytunnels) (3.28 kg m^−2^, n = 19) (Figure 7). Surprisingly, the yield found within controlled-environment spaces was the lowest (2.65 kg m^−2^, n = 508); this result will be discussed. Looking specifically at certain papers may aid understanding of this section, especially as both greenhouses and controlled environments encapsulated 94.3% of observations. These values are then difficult to interpret as they are broad and will absorb a lot of the between-study heterogeneity seen in this study. Seasonal impacts were also evaluated. Winter (8.93 kg m^−2^), was much higher yielding than spring, summer, and autumn, which all yielded 5.11, 3.95 and 3.43 kg m^−2^, respectively. 

When evaluating the interaction between time and season, we also discovered that their interaction accounts for ~37% of the heterogeneity observed. This value is interesting, especially as chi-squared results indicate that there were 2.5 more iceberg lettuces cultivated in winter, a high value that will be interpreted and discussed. 

#### 2.4.5. Impact of Lighting Types (n = 979)

**Figure 8:**
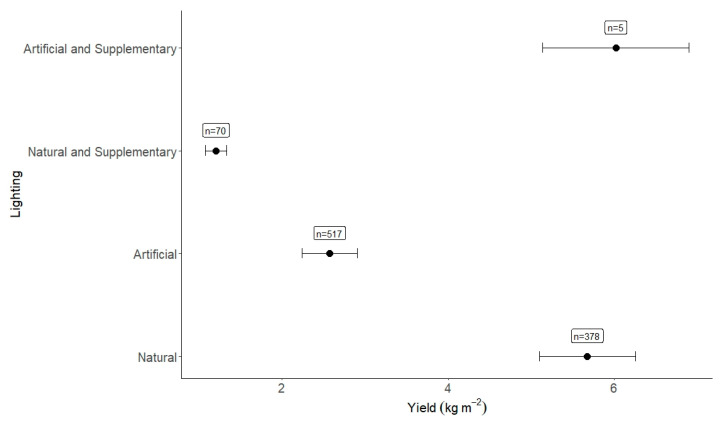
Yield per lighting type. The number of observations is given alongside the effect size result.

There seems to be a weak relationship between lighting type and yield; in meta-regression, it accounts for ~12% of the heterogeneity seen in this study. Results from the lighting section are interesting as they generally tell contrasting stories (Figure 8). ‘Artificial AND supplementary’ light yielded the highest (6.02 kg m^−2^, n = 5), followed by natural lighting (5.68 kg m^−2^_,_ n = 378). As supplementary lighting to natural light did not increase the yield as with artificial lighting situations (conversely, it reduced it substantially), it appears that there is no significant relationship between lighting type and yield. A meta-analysis conducted on individual lettuce cultivars has revealed that natural lighting is most effective for all lettuce cultivars in this study, except for one cultivar type (cos), which was trialed with artificial and supplementary lighting. The result for cos lettuce under artificial and supplementary lighting (6.02 kg m^−2^) is much higher than the overall global analysis cos result (2.70 kg m^−2^).

#### 2.4.6. Impact of Water and Nutrient Delivery Technologies (n = 967)

When looking at the overall watering type used, there was a high proportion of hydroponic studies, which dominated the analysis, making up ~90% of all observations. The results show that aquaponics was the highest-yielding system (6.73 kg m^−2^, n = 50). This was followed by soilless culture (systems that were potted but using an inert substrate (5.70 kg m^−2^, n = 38), and then hydroponics (3.43 kg m^−2^, n = 881). Aeroponic technology was also used (2.40 kg m^−2^, n = 10), but was only included in one study that satisfied the inclusion criteria.

**Figure 9:**
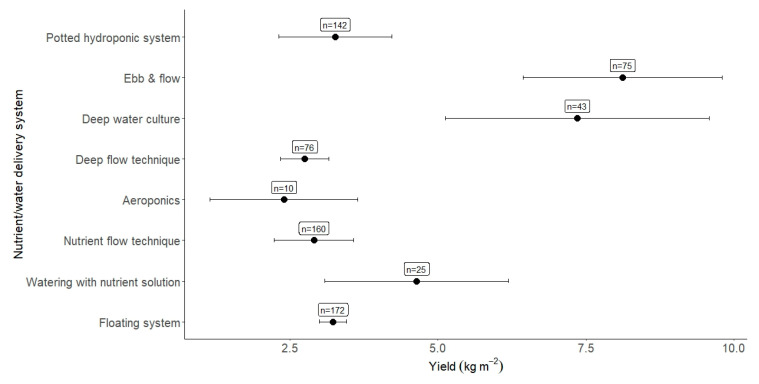
Yield per nutrient/watering system. The number of observations is given alongside the effect size result.

A breakdown of specific technologies utilised within the overall watering type was also evaluated, giving a breakdown of individual watering/nutrient delivery technologies (Figure 9). By completing subgroup analysis on the category of watering type, we discovered that ebb & flow yielded the highest returns (E&F, 8.12 kg m^−2^), followed by deep-water cultivation (DWC, 7.36 kg m^−2^). Watering and nutrient delivery types accounted for ~22% of the heterogeneity observed.

#### 2.4.7. Evaluating the Fit of the Model

The Akaike Information Criterion (AIC) model selection was chosen to distinguish between the independent variables in the overall meta-analysis, describing the relationship and importance of the season, watering and nutrient types, cultivar, lighting and building type on the yield of lettuce within CEA systems. The best-fit model, carrying 69% of the cumulative model weight with an R^2^ value of 82.28%, included the interaction between season, nutrient delivery system, cultivar and lighting. The R^2^ value represents the percentage of heterogeneity in the meta-analysis explained by the model. The second-best-fit model, carrying 31% of the cumulative model weight, included the same interactions as the best-fit model but with a building type that was only 1.62 AIC units higher. The second-best-fit model did have a higher R^2^ value (82.29%), though the use of an extra explanatory variable for only a 0.01% increase in observed heterogeneity indicates that the impact of the additional variable is negligible.

This AIC model selection has highlighted the variables with the highest level of impact on the model and hence the outcome of lettuce yield within CEA systems. The analysis found here has highlighted the nutrient delivery system as the most important factor (almost halving R^2^ when removed from the model), followed by the season, the cultivar examined and then the building type. All factors are important, but this study found the four factors in the best-fit model created the lowest AIC and hence explained the observed variation from the data best, reducing the number of variables that have a significant impact on the observed heterogeneity in the meta-analysis. Other factors also did have an impact (notably time and building type), but as mentioned above, when included in models, the addition of both variables only increases R^2^ by 0.19%, not significant enough for the parameters to be included in the best-fit model as the increase is so small for the amount of extra information the model has to evaluate and process for a slightly better explanation of model heterogeneity.

### 2.5. Key Results

- About 121 papers were evaluated, totaling 979 observations within those studies.
- About 85% of all papers were from between 2017 and 2022.
- Mean yield from the global analysis was 3.68 kg m^−2^, much higher than the global FAO field value of 1.88 kg m^−2^ [14].
- Mean yield from vertically grown lettuce was 6.88 kg m^−2^.
- Average time to harvestable size for this study was 40 days, with 94% of papers having trial periods of 70 days or less. The time needed is considerably lower than typical field-based cultivation (60 to 120 days) and will be discussed [13].
- Iceberg lettuce was found to be the highest-yielding variety, reaching on average 7.45 kg m^−2^. Variety will be discussed as there are many varieties of lettuce, but the variety is an independent variable; it does not drive the different changes, even if its characteristics do influence yield.
- Greenhouses were found to be the highest-yielding building type (5.11 kg m^−2^), with the season of winter also yielding the highest (8.93 kg m^−2^).
- Aquaponics was the highest-yielding system (6.73 kg m^−2^). For comparison, the most common watering type of hydroponics yielded 3.43 kg m^−2^.
- Ebb and Flow was discovered as the highest-yielding subgroup watering/nutrient delivery method (8.12 kg m^−2^).

## 3. Discussion

### 3.1. Systematic Search and Study Descriptions

Overall, 121 papers were used in this study, totaling 979 observations. Due to data limitations, this meta-analysis had to omit 113 papers, resulting in another 955 observations being excluded. As a result, certain variables were not able to be fully interrogated; for example, an initial 50 observations for aeroponic technology were reduced to only 10 observations from a single study (due to a lack of key data for use in meta-analysis, i.e., no error terms). This lack of data limits the power of this analysis, especially as a number of studies have found superior performance of aeroponic systems compared to hydroponics, with some studies observing between 50 and 100% higher yields [13,15].

This study also discovered how widespread research in this field is. From the whole dataset, there were 36 different nations that completed research, coming from all continents globally (Figure 1). Even though papers came from so many different nations, it was interesting to note that 66% of papers came from the five nations with the most observations, indicating there are certain nations that have a high output of research papers, such as the USA, China, Italy, Japan and Brazil. The most produced lettuce varieties were iceberg and cos, of which iceberg accounts for between 56 and 74% of all lettuce produced in Spain and the USA, with cos accounting for ~25% of production in both nations, making them two of the largest global producers of lettuce [16]. 

The global analysis, encompassing all studies, showed CEA lettuce yields are two times that of field-grown lettuce (3.6814 kg m^−2^ vs. 1.8773 kg m^−2^) [14]. This value from the FAO is from the same period as the meta-analysis results (2009–2022) and includes results from all nations in their database. The result from the FAO includes all field-based cultivation globally and will contain places that may not be optimal for lettuce production. When comparing the USA’s and Spain’s field production yields (3.43 kg m^−2^ and 2.83 kg m^−2^, respectively), we see that CEA production does offer higher yields but not as significant an increase as against the FAO’s global average [10]. The global result indicates that CEA lettuce can increase yields over field-grown lettuce, and in the literature, this is also apparent. 

Though the results of the global analysis are statistically significant, they are marred by heterogeneity; hence, caution should be taken when establishing their interpretability. An example of this high heterogeneity is exemplified by Touliatos [17] in comparing vertical farming yields to those of conventional horizontal farming. The shoot fresh weight per plant in their study favoured horizontal production; however, the yield per occupied growing floor was around 14× higher in the vertical farming system (95 kg m^−2^ vs. 6.9 kg m^−2^), considerably higher than any form of horizontal production result in this study [17].

Though the results from both conventional CEA production and vertical production met the inclusion criteria, many of the vertical farming values had to be excluded from the global analysis due to how much they skewed the data. Papers, where plants were grown in a vertical farm or plant factory, were generally grown in two-layered growth chambers. The meta-analysis of all suitable vertical studies has shown an average yield of 6.88 kg m^−2^, with quite a large range of results. 

### 3.2. Sub-Group Analysis Discussion

#### 3.2.1. Impact of Time and Season

The average time to harvestable size for this study (40.4 days) was much quicker than current production, which ranges between 65 days from the beginning of germination to harvest for warmer months and up to 120 days for winter-planted lettuce [13]. The correlation result between the time taken from germination to harvest is an interesting result, especially as Pearson’s correlation coefficient does not equal one. Previous work on the relationship between yield and time taken indicates that lettuce has a slower initial growth phase, which then exponentially increases until the senescence point, being influenced by other parameters such as planting density and water availability [18,19,20]. The use of many papers testing different cultivars under different conditions influences the correlation result. If all plants were from one homogenous population, then the correlation relationship between yield and time would be equal to one, but external factors influence plant growth rate, which does not allow linear growth rates. 

As mentioned above, a selection of different lettuce varieties was used in the experiments evaluated in this meta-analysis. From the results, iceberg cultivars yielded the highest (7.45 kg m^−2^), whereas looseleaf lettuce yielded the lowest (2.58 kg m^−2^). Types of lettuce cultivars vary greatly in the time they need to reach peak maturity. Research has found that iceberg lettuces are the highest-yielding cultivar, but on average they take the longest to reach harvestable size, with looseleaf varieties reaching peak maturity much sooner but with lower yields [21]. This was reflected in this meta-analysis, where icebergs have the largest yield but also, on average, take the longest time to reach harvestable size.

In this meta-analysis, varieties were separated into individual groups to remove the influence of each variety’s characteristics. As highlighted in the results, the cultivar itself had minimal impact on the pattern of results seen in this study, though it must be recognised that different lettuce varieties did grow for varying periods of time and reach different harvestable sizes; hence, actual yield values will vary between each variety. But as seen in Appendix SF, they all follow a similar pattern that follows that of the global meta-analysis (i.e., ‘greenhouse’ being the most effective building type for almost every lettuce variety and ‘other’ the least effective), showing that variety has not impacted the results, just that yields vary due to other characteristics the lettuce variety has, such as the aforementioned length of cultivation. 

Due to lettuce’s short vegetation period and ability to withstand cold weather, it can be cultivated in any season [22]. This meta-analysis evaluated the season as a factor for crop growth, especially as many studies in CEA still use natural lighting over artificial lighting for trials in greenhouses. As seen in the results, winter was much higher yielding than any other season; it was the highest-yielding season for all lettuce varieties except for looseleaf (where the highest was Autumn). When analysing the impact of the growing season on yield, its relationship with lettuce variety was evaluated, finding a relationship between both factors. When performing a chi-squared test on the relationship between season and variety, there were 2.5 and 1.5 times more experiments on larger varieties (iceberg and multi-leaf, respectively) than would have been expected in this study in Winter. When evaluating the relationship between these factors, they accounted for ~37% of the heterogeneity in this study, which combined represents a very large proportion of the heterogeneity observed. This result is a touch surprising, as lettuces are well known to perform better in warmer seasons, providing optimal temperatures and higher levels of irradiance to ensure consistent crop growth [23]. However, nearly 75% of studies performed in winter were conducted with natural lighting and within a greenhouse, the two highest-yielding options of their respective subgroup variables. Suboptimal temperatures can have a serious effect on crop yield, with temperatures too low allowing no plant growth and too high causing heat stress and crop spoilage [24,25]. Different varieties necessitate slightly different conditions, and certain seasons may be more appropriate than others, especially as lettuce varieties exhibit diverse behaviours, particularly obvious when exposed to too high temperatures and solar radiation [26]. Despite this, the ecological requirements for lettuce are fairly forgiving, yet its physiology is still significantly influenced by growing conditions.

#### 3.2.2. Impact of Water and Nutrient Delivery Systems

In this meta-analysis, hydroponics dominated the water delivery systems in terms of research performed using the technology, but for CEA lettuce growth, aquaponics was identified as the highest yielding. Hydroponics is the most established technology utilised for water and nutrient delivery within these systems, yet aquaponics is now being considered as having great potential as an organic production method for both vegetables and aquatic organisms, with the nutrient-rich water from the aquatic organisms being used for plant growth [27]. Nutrient supplementation from aquaponics, where standard hydroponic nutrients are supplemented by organic nutrient fertilisers from aquaponics, is the key reason why it has increased yield over standard hydroponics and the inorganic nutrient solutions typically used [28]. Aquaponics has been found to equal or better hydroponics, ranging from an 8% to 39% increase in yield. It has also been found that aquaponic solutions can achieve similar plant yields at lower nutrient concentrations, lower conductivity and lower pHs than hydroponic systems [24,25]. The literature’s findings are significant and indicative of aquaponics higher yields, yet they are still significantly lower than this meta-analysis average of a nearly 100% increase in yield between the two technologies.

Aquaponics was also highlighted in the literature as a promising technology due to the multiple other benefits it provides. Castillo-Castellanos’ [27] paper stated that though aquaponics in his study were slightly lower in yield due to nutrient balance problems, they were still more profitable than hydroponics due to the fish produced. Monsees [29] also highlights the benefits of aquaponic systems, claiming that even with nutrient supplementation in aquaponic systems, mineral fertiliser savings could be as high as 63%, reducing lettuce emissions by 72% overall.

This meta-analysis also analysed specific water and nutrient delivery technologies, highlighting ebb & flow (E&F, 8.12 kg m^−2^) and deep-water cultivation (DWC, 7.36 kg m^−2^) as the highest-yielding technologies. When comparing the overall water/nutrient delivery systems (i.e., hydroponic, aeroponic, soilless, and aquaponic), we see that floating systems (n = 172) and nutrient flow technique (NFT) systems (n = 160) were the most utilised. Interestingly, though DWC and E&F were the highest-yielding systems, 70% of DWC papers came from the overarching hydroponics category, which overall yielded under half of DWC on average. E&F and DWC had the highest results, just under 3 kg m^−2^ higher than any other sub-system.

When looking at the literature, there is little difference in the efficacy of these watering/nutrient systems, with a number of studies finding minimal differences in yields between them. Other factors were found to be important also, notably with NFT systems, which tended to yield lower due to low levels of root contact with nutrients and water but are still employed due to their higher water use efficiencies when compared to other technologies and field-based cultivation [30,31].

The literature was reflected in the variety of sub-group meta-analyses; each variety preferred a different watering system. In general, where aquaponics was an option, that was the highest yielding; otherwise, it was soilless culture (which involves traditional aerial watering), followed by hydroponics. Hydroponics dominated this study in terms of observations (n = 881/979) and hence absorbed a lot of heterogeneity. For the nutrient delivery systems, each variety preferred a different watering system; hence, more research is needed into the right system for each variety. The highest-yielding subsystems (E&F and DWC) were also both mainly cultivated within greenhouses (66% and 63%, respectively), as well as with natural lighting (66% and 41%, respectively), explaining the high yields found from those two watering/nutrient delivery systems.

As well as yield and water use efficiency, parameters such as nutrient efficiency, operating cost, and the amount of management needed are all parameters that should influence the choice of technology [32]. For watering systems, choosing between a recirculating and non-recirculating system is also essential; nutrient and water recycling from recirculating systems will help reduce the environmental impact of the system due to resource savings.

Aeroponic technology was included in this analysis, but due to a few observations, it is difficult to interpret its efficacy and potential. Aeroponics is the most novel of the water delivery technologies and is still yet to be widely used due to high investment and management costs [33], most likely explaining the lack of papers on the topic of lettuce cultivation. Yields from aeroponic systems are promising, as non-submerged, exposed root systems can provide easier access to carbon dioxide for roots as well as reduced water and nutrient consumption when compared with other hydroponic technologies [34].

#### 3.2.3. Impact of Lighting

The lighting results indicate there is no significant relationship between lighting type and yield in this meta-analysis. Overall results have indicated that artificial lighting with supplementary lighting is the most effective within CEA systems, but this was based on one experiment with five observations, with natural lighting yielding only 0.34 kg m^−2^ less but with a much larger sample size (n = 378). This meta-analysis only encapsulated overall lighting type and did not focus on photoperiod, breakdown of lighting type (fluorescent or LED), lighting intensity or lighting quality. These other lighting parameters have more of an effect on successful plant development, especially lighting spectra/quality and lighting intensity [35].

Literature consensus is split on which lighting quality is most effective for CEA lettuce yield: full-spectrum white light or red-blue light. It is well established that combinations of red and blue light are especially effective in encouraging plant growth and development and are more reliable and efficient than full-spectrum lighting [36].

Experimental studies that determined optimal light spectra found ratios between 3:1 and 5:1 red-blue lighting to be ideal for lettuce cultivation [36,37,38,39]. Alternatively, some studies found white full-spectra light to be best for rendering higher yields, indicating white lighting performed better and had lower energy consumption [40]. White light contains all light spectra, including green and far-red, which have also been found to have beneficial traits that can regulate photosynthesis and plant morphology [41]. Fraszczak’s [42] paper, though, had interesting outcomes, demonstrating that cultivars of different colours required different lighting combinations to ensure optimal growth.

When looking at the impact of supplementary lighting, it is generally seen to increase yields in CEA systems, especially within vertical systems, yet within this study, supplementary lighting was found to increase yields in systems with artificial light already but not within natural light systems. Studies have found supplementary lighting of either red-blue or white light to be effective in increasing yields, with the main downward lighting being white light [43]. Supplementary lighting is beneficial and should be utilised within these systems as it helps extend the photoperiod, but more research is also needed to determine the optimal duration of supplementary lighting [44].

Many studies found that longer photoperiods at lower light intensities (ppfd ~230–260, period of 16/8 h or 18/6 h) increase yield over higher light intensities with shorter light periods [45,46,47]. Papers comparing lighting types also consistently found that LED lights (over fluorescent and metal halide, for example) provide many benefits, notably in their energy savings and easily manipulable lighting spectra [48,49,50]. LEDs also run at lower temperatures than previous lighting types, reducing heat waste, ensuring less water loss, and increasing the sustainability of the system [51]. Other factors were also investigated within studies, notably ensuring uniformity of lighting to guarantee unvarying growth within your system, which was highlighted as important, especially for VF systems with multiple stacked layers of growth [52,53].

#### 3.2.4. Other Influencing Factors

Building type results from this study suggest greenhouse cultivation as the highest yielding. Greenhouse studies in this meta-analysis were often conducted in ‘high-sun’ locations (81%), which explains the high yields within greenhouses compared to other building types. This study included mainly single-layer cultivation, and due to outlier points, data were excluded as they heavily skewed the analysis. Greenhouse cultivation is beneficial and, in many circumstances, more sustainable than indoor controlled environments with artificial lighting, as they often utilise natural lighting [54]. As mentioned in the results, the lowest-yielding building type was controlled-environment spaces. A controlled environment was the most commonly used building type in the analysis (n = 508) and hence absorbed a large amount of between-study heterogeneity. Trials, though within controlled-environment spaces, were performed mostly on the four lowest-yielding lettuce varieties (88%), as well as having shorter trial times than the average (35.5 days), explaining the lower yields from this building type. Stacked vertical farming, as stated earlier in the discussion, can quickly increase yields over the same land area, driving up efficiency and yields simultaneously. As mentioned, this study found that vertical systems can improve yields per area, producing over 3.5 times the yield seen in the field (6.88 vs. 1.88 kg m^−2^). Many of the studies used growth chambers with only two growing shelves, hence a relatively small yield increase as compared to single-layer cultivation, but there were studies with higher planting density and more stacks yielding much higher than the average. Touliatos’ [17] paper, for example, was a side-by-side trial of vertical vs. horizontal cultivation and highlighted that vertical yields were up to 14× higher. This is game-changing for agriculture, especially as it helps to tackle waning global agricultural land issues partially caused by business-as-usual field-based cultivation but also highlights some of its advantages over other CEA growing methods. Researchers have been working on trying to improve the landscape of vertical growing compared to horizontal, with this analysis finding studies that stretched from improving lighting conditions to building automated measurement systems to evaluate plant growth in real-time, demonstrating the breadth of research performed in this field [25].

Other interesting studies also arose from the literature search, notably Moreno-Perez [55] on nutrient recycling, highlighting that yields were similar between systems with or without nutrient solution recycling, the benefit being a more efficient use of water and nutrients. Nutrient use within papers was evaluated by many studies (n = 429), with research evaluating nutrient deprivation, organic vs. inorganic nutrient solutions, nutrient solution concentration, and composition [30]. Aquaponics studies, though, have really highlighted the potential benefits of aquaponic solutions for CEA plant growth, especially in terms of nutrient and fertiliser savings, which notoriously have high emissions associated with fertiliser production [56]. The nutrient solution throughout the papers tended to be either standard Hoagland’s solution or a variation of it.

As understood from Frasetya’s [57] paper on evaluating nutrient formulations in the growth of lettuce, it is crucial for the quality of lettuce to be high to be marketable. The nutritional quality of lettuce crops was also a theme of importance across studies, with researchers understanding that the nutritional quality of lettuce is one of its most valuable assets and makes it an attractive food source [30]. CEA-based cultivation of lettuce needs to ensure that its crops are at least as nutritionally valuable as field-based crops. Different lettuce cultivars have overall nutritional value differences, with microgreens and leaf varieties of lettuce having higher concentrations of micronutrients, mainly due to head lettuce having a high closure of leaves [22].

A final consideration not evaluated within most papers included in this study (or this study itself) is the environmental cost of CEA systems as compared to open-field, soil-based production. Without renewable energy sources to power CEA systems, the environmental impact of the system can be very high, up to 17.8 kg CO_2_ eq. kg^−1^ lettuce, larger than the 10 kg CO_2_ eq. kg^−1^ lettuce associated with intercontinental air freighted transport of field-based systems [58]. However, soilless closed systems can yield orders of magnitude more produce per m^2^ and may not require any additional net land occupation as they can be integrated into existing infrastructure and buildings. When factoring in the carbon opportunity of land use, closed systems can produce a smaller carbon footprint than most field supply chains at 0.48 kg CO_2_ eq. per kg^−1^ of lettuce (however, this would also only be the case with renewable energy sources) [58].

## 4. Materials and Methods

Meta-analysis is a statistical method that combines and synthesises multiple studies and integrates their results, increasing the overall sample size and thereby improving the statistical power of the analysis [59]. This meta-analysis will primarily look at the yield of lettuce crops within CEA systems, as it is the most studied and commercially prevalent crop within CEA. To obtain this, a systematic search and meta-analysis will be completed to encapsulate all relevant papers and then synthesise the data. CEA lettuce yields will be compared to field-grown lettuce to understand the potential expected benefits of CEA.

### 4.1. Systematic Search 

Standard systematic search methodologies were followed [60,61,62] to collate empirical data on lettuce growth within CEA systems. A time constraint was placed upon the search, allowing papers from 2009 onward to capture only the most relevant recent papers.

**Figure 10:**
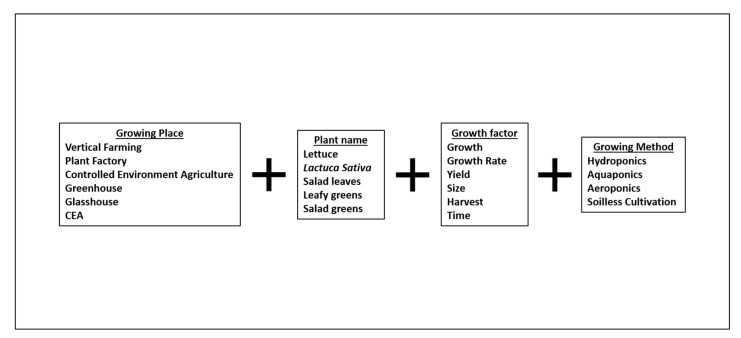
Search terms for systematic searches. Terms also included variations on each phrase (i.e., Vertical farming will include searches of “Vertical farm”, “Vertical farming” and “Vertical farms”). A detailed breakdown of the systematic search is available in Appendix SD.

A structured search string was used to ensure that all relevant literature was captured without bias (Figure 10). Boolean operators and truncations were used where possible in the search. Using the search terms in Figure 10, 2880 individual searches were completed across four databases (720 each): Science Direct, Web of Science (WoS), MDPI and Scopus. To ensure meaningful comparisons, publications had to satisfy strict inclusion criteria. These were as follows:

1. The metrics necessary for meta-analysis: mean, sample size and error term (standard deviation);
2. Studies from 2009 to 2022 (12 February 2022 acceptance date);
3. Papers containing primary quantitative data;
4. From a scientific, peer-reviewed journal;
5. Studies must be in English or translated into English.

Data from the publications were extracted, with standard conversions taking place where necessary to ensure all the data is in the predefined unit (kg m^−2^). Where data within papers was lacking or insufficient for analysis, authors were contacted for data. Assumptions of germination times were completed for papers that include germination times in reference to the quantity of leaves at the transplanting stage (Appendix SE). Many studies reported data through figures only, so numerical information was extracted using Plotdigitizer [63]. 

### 4.2. Meta-Analysis

The meta-analysis was completed in R version 4.1.2 [64], using the ‘META’ and ‘METAFOR’ packages [65,66]. Effect size calculation (See Appendix SF) was determined using the ‘METAMEAN’ function [63]. Random effects models were applied to calculate the overall effect sizes for the global analysis but also for the following interactions between yield and influencing variables: time, lighting type, nutrient delivery system, building type and lettuce variety. 

Studies varied greatly in how yields were manipulated and influenced, depending on multiple factors such as lettuce variety or the nutrient delivery system used. As we anticipated considerable between-study heterogeneity, a random effects model was used to pool effect sizes. The random-effects model assumes that there is not only one true effect size but a distribution of true effect sizes [15]. It allows for different study-specific effect sizes, assuming that the effect varies not only due to sampling error but also due to heterogeneity between studies, with the pooled mean representing a random sample of a relevant distribution of effects [15]. The meta-analysis was weighted using the inverse variance method model, employing the restricted maximum likelihood estimation (REML) estimate. Heterogeneity was evaluated using the Q and I^2^ measures, and the REML estimate was selected to estimate Tau^2^ (T^2^) values.

Subgroup analysis was conducted on the categorical variables extracted from each study: lettuce variety, building type, season, country, nutrient distribution system, and lighting. Where data were missing for specific subgroup analysis (i.e., where building type was not included in the paper), the study was removed and a new random-effects model was run for each subgroup parameter, but with a reduced sample size due to the removed papers. This provided subgroup analysis results that were not skewed by missing data points. Results from subgroup analyses were then extracted from R and plotted. Meta-analyses were also run by subgrouping the lettuce varieties to account for the potential influence that different varieties may have, considering they have different characteristics and reach different harvestable sizes. Vertical systems were also subgrouped and evaluated, as the large values observed skewed the data too much. A meta-regression was conducted to determine the relationship between effect size and many of the continuous independent and categorical variables, as well as the interactions between the variables themselves: building type and season, watering type and watering/nutrient delivery method. Meta-regression was performed employing a mixed-effects model, which accounts for the fact that observed studies deviate from the true overall effect due to sampling error and between-study heterogeneity [15]. The Akaike Information Criterion was also used to determine which variables within the model are important for predicting the relationship between lettuce yield and the many independent variables. To obtain this, several possible models were constructed with different numbers of independent variables, which were then compared using AIC.

## 5. Challenges Faced and Future Study Recommendations

There were also many challenges faced, primarily the failed reporting by published studies of important data for completing meta-analyses. More care needs to go into reporting key trial data, especially reporting values of variance or standard error. There were 233 studies initially, but due to missing data, over 100 of them had to be excluded. Future work in this field should hopefully build upon this analysis to try and further understand all the factors that influence lettuce yields within CEA environments. Further study should include review studies on individual subgroup parameters to understand in greater depth the comparative performance between certain technologies within CEA systems.

## 6. Conclusions

Overall, this study has consolidated many of the research outputs from controlled-environment agriculture studies on lettuce growth. This meta-analysis contained high levels of between-study heterogeneity, which to some extent limits the interpretability of the results found. CEA plant development has attracted lots of keen research interest, and a comparison of the most effective technologies within these systems has been needed to understand what factors influence growth most.

From our study, the water/nutrient delivery system was the most influential on yield, and key research needs to be performed to further understand which is the most efficient system.

Overall, from this study, the main outcomes are:

- Lettuce from CEA systems yields on average double that of field-based cultivation and has quicker production rates (50% faster in summer periods and up to 300% faster in winter).
- Different cultivars of the same plant need different conditions, which all must be individually accounted for. There is not a ‘one size fits all’ recipe for lettuce growth within these systems. Detailed research into the conditions needed by each cultivar is required. Aquaponic systems resulted in the highest yields, with their organic nutrient solutions able to achieve similar plant yields but at lower nutrient concentrations, pHs and conductivities.
- Model selection has revealed that the season, nutrient delivery method, cultivar and lighting type are the most influential variables within this model, explaining ~70% of the observed variation in the data. Building type and time were also found to be influencing variables, yet the nutrient delivery method contributed most towards explaining heterogeneity, having the largest influence on lettuce yields in this meta-analysis. More research into nutrient delivery methods is needed, but understanding seasonal impacts is also necessary, especially for greenhouse or protected outdoor production.
- Ebb & Flow was discovered as being the highest-yielding nutrient delivery system, but from reviewing the literature and analysing the efficacy of individual nutrient delivery systems in this meta-analysis, there is little difference in the efficacy of these nutrient delivery technologies; it comes down to preference. Supplementary lighting is beneficial for plant growth, but there is still no consensus on which spectrum is most beneficial for increasing yields in CEA systems.
- Greenhouse yields were the highest of any building type, with natural lighting as its primary light source producing some of the highest yields in this meta-analysis. Greenhouse cultivation is beneficial, especially when considering the environmental impact of fully controlled-environment systems whose artificial lighting and climate control systems necessitate considerable energy input.
- Vertical growth provides much higher yields per area than horizontal single-layer cultivation and field-based cultivation (6.88 kg m^−2^). Further research into commercial vertical farms is needed, as many studies have used two-layered growth chambers, which are not truly representative of the potential vertical farming has for increasing yields per area.
- Aeroponics is a promising technology, but a lack of data has limited this study’s ability to infer its efficacy and potential. More research is needed to understand its yield potential against other water/nutrient delivery technologies.
- Other factors, such as taste and appearance, are just as important to consumers and need to be considered for lettuce growth as well. The environmental impact of CEA systems also must be considered, as without renewable energy sources, the impacts can be higher than those of conventional, field-based systems.

## Figures and Tables

**Table 1:** Data extraction parameters for meta-analysis, including standard units for measurements. Further explanation is available in Appendix SA.

**Parameter**	
Paper ID	Yield per plant (g)
Study	SD (g)
DOI	Area (m^2^)
Title	Time (days)
Author	Planting density (plants m^−2^)
Year	Lettuce Variety
Season	Overall watering system (i.e., Hydroponic, Aeroponic, Aquaponic)
Country	Nutrient delivery system (i.e., Ebb & Flow, Deep water culture)
N	Building type
Mean (kg m^−2^)	Lighting type
SD (kg m^−2^)	

## Data Availability

Data sharing is not applicable.

## Supplementary Materials

The following supporting information can be downloaded at: https://www.mdpi.com/article/10.3390/plants12142623/s1, Appendix SA: Full systematic search methodology; Appendix SB: Germination times; Appendix SC: Effect size calculations; Appendix SD: Experimental factors assessed and data extraction parameters; Appendix SE: Heterogeneity plots and results; Appendix SF: Variety subgroup result; Appendix SG: Author list.
