# Supplementary material for: Let-Us Investigate; A Meta-Analysis of Influencing Factors on Lettuce Crop Yields within Controlled-Environment Agriculture Systems
Source: Plants (Basel). 2023 Jul 12;12(14):2623. doi: 10.3390/plants12142623 (PMC10385570; doi:10.3390/plants12142623)
Supplement: Supplementary file 1 [file plants-12-02623-s001.zip › plants-2500938-supplementary.pdf]

‘Let-us investigate; A meta-analysis of influencing factors on lettuce crop yields within CEA systems.’
Michael Gargaro, Richard J. Murphy, Zoe M. Harris

Appendix A

Table S1: List of all experimental factors utilised with studies in this meta-analysis. Papers often covered more than one factor.

Experimental factors assessed within studies
Aeration
Cultivar
Cultivar and Lighting
Growth environment
Growth mediums
Growth mediums and Nutrients and Season
Lighting
Nutrients
Nutrients and Aeration
Nutrients and Cultivar
Nutrients and Growth mediums
Nutrients and Lighting
Nutrients and Temperature
Nutrients and Watering
Season
Season and Nutrients
Watering
Watering and Cultivar

In this section, I will also expand on Table 1 from the paper, and explain some of the parameters extracted from suitable studies, and what each parameter encompasses. Most are self explanatory (such as Author name) but others I will provide a brief statement explaining them for clarity.

Table S2. Data extraction parameters for meta-analysis, including standard units for measurements

Parameter	
**Paper ID**	**Ordered number value assigned to each paper**
**Study**	**Value assigned to each individual observation.**
**DOI**	**The DOI of the paper to identify it.**
**Title**	**Title of the paper.**
**Author**	**Primary Authors name**
**Year**	**Year the paper was published in**
**Season**	**The season in which the trial took place. Different for different regions.**
**Country**	**The country where the experiment took place, not where the paper was necessarily published from.**
**N**	**Number of plants in each trial.**
**Mean (kg m^{-2})**	**Lettuce yield.**
**SD (kg m^{-2})**	**Standard deviation of the above lettuce yield.**
**Yield per plant (g)**	**Yield of each individual lettuce in the observation.**
**SD (g)**	**Standard deviation for each individual plant yield.**
**Area (m^2)**	**The planting area.**
**Time (days)**	**Time taken for the trial, planting to harvest.**
**Planting density (plants m^{-2})**	**Planting density in the study.**
**Lettuce Variety**	**Which lettuce variety/cultivar was investigated.**
**Overall watering system (i.e Hydroponic, Aeroponic, Aquaponic)**	**The overall watering system used**
**Nutrient delivery system (i.e Ebb & Flow, Deep water culture)**	**The specific nutrient delivery technology chosen. Technologies within this category are linked with the overall watering system above. For example, Ebb & Flow could be part of either Hydroponics or Aquaponics.**
**Building type**	**Building type the trial took place in.**
**Lighting type**	**The lighting type utilised in the study.**

Appendix B

Heterogeneity plot for the global analysis (funnel plot), and printed results of Q, I^2 and H^2.

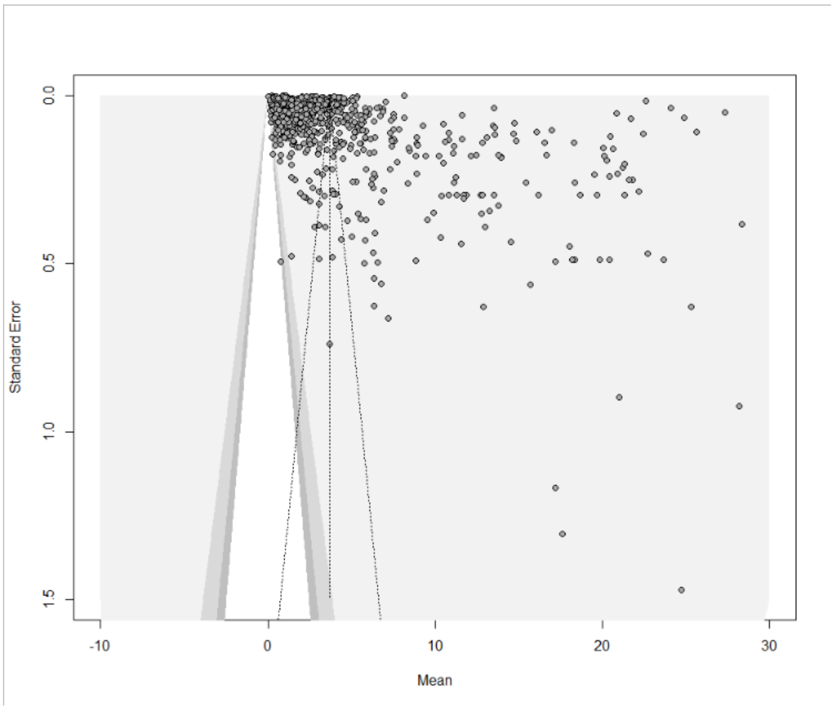

Figure S1: A funnel plot for the global analysis

```
Random-Effects Model (k = 979; tau^2 estimator: REML)

tau^2 (estimated amount of total heterogeneity): 23.0514 (SE = 1.0442)
tau (square root of estimated tau^2 value):      4.8012
I^2 (total heterogeneity / total variability):   100.00%
H^2 (total variability / sampling variability):  513680.32

Test for Heterogeneity:
Q(df = 978) = 289285629.8168, p-val < .0001

Model Results:

estimate      se     zval    pval   ci.lb   ci.ub
  3.6814  0.1536  23.9683  <.0001  3.3803  3.9824  ***

---
Signif. codes:  0 ‘***’ 0.001 ‘**’ 0.01 ‘*’ 0.05 ‘.’ 0.1 ‘ ’ 1
```

Appendix C

An example of the subgroup results seen for building type from each individual lettuce variety meta-analysis.

Similar patterns across all varieties. Greenhouse is the highest yielding for the majority of lettuce varieties. This is an example variable which represents the overall pattern seen for all varieties across all independent variables.

Iceberg

```
Results for subgroups (random effects model):
                             k    mean              95%-CI
bt = Greenhouse             19 13.8390 [11.1055; 16.5724]
bt = Outdoor covered         4  2.1259 [ 1.2761;  2.9758]
bt = Controlled environment 18  1.9299 [ 1.4674;  2.3925]
```

Cos

```
Results for subgroups (random effects model):
                             k   mean            95%-CI
bt = Controlled environment 73 2.5169 [2.0778; 2.9560]
bt = Greenhouse             49 3.1745 [2.3621; 3.9869]
bt = Other                   7 1.2527 [0.4984; 2.0071]
```

Batavia

```
Results for subgroups (random effects model):
                             k    mean              95%-CI
bt = Controlled environment 91  3.0806 [ 1.9589;  4.2024]
bt = Greenhouse             60  3.0714 [ 2.6746;  3.4682]
```

Butterhead

```
Results for subgroups (random effects model):
                              k   mean            95%-CI
bt = Controlled environment 107 2.7646 [2.4448; 3.0844]
bt = Greenhouse              93 3.1853 [2.1384; 4.2321]
bt = Other                   11 1.6920 [0.9474; 2.4367]
bt = Outdoor covered          6 3.6202 [2.9522; 4.2882]
```

Looseleaf

```
Results for subgroups (random effects model):
                              k   mean            95%-CI
bt = Greenhouse              74 4.7441 [3.5098; 5.9784]
bt = Controlled environment 177 1.6999 [1.4599; 1.9399]
bt = Outdoor covered          7 2.6991 [1.9932; 3.4050]
bt = Other                    8 1.8149 [1.2387; 2.3911]
```

Multileaf

```
Results for subgroups (random effects model):
                             k   mean             95%-CI
bt = Controlled environment 34 6.6345 [3.3841;  9.8850]
bt = Greenhouse             10 7.8524 [3.7311; 11.9738]
```

Appendix D

Full Systematic search Methodology

The initial search was conducted on 12 February 2022, developed by Michael Gargaro for this analysis. The methodology for the systematic review was taken from Koutsos’s [60], a well cited paper focussing on systematic literature reviews within agricultural sciences. Methodological inspiration has also been taken from another well cited paper by Pullin and Stewart [61], focussed on systematic reviews in Environmental management and Mengist’s [62] paper on systematic literature review and meta-analysis for environmental science research.

The searches were conducted using four commonly used databases; Web of Science, Science Direct, MDPI and Scopus. The search was conducted in these various internationally recognised databases to collect all relevant information, ensuring that all publications that fall within the search criteria were encapsulated. This should ensure that the search was exhaustive.

An initial pre-scoping search was completed in December 2021, utilising the four final databases as well as Google Scholar. The trial included only three categories of search term, “Plant name”, “Growth factor” and “Growing method”, of which the final category also included terms now separate, in “Growth place”. In the final methodology, four overall categories were utilised to specify the search (Figure 1 in main text).

In this initial trial, only the number of hits were registered, giving a rough number of papers to expect. For the first search string completed (“Vertical Farming” AND “Lettuce” AND “Growth”), it was clear that Google scholar returned a much higher number of papers, returning 942 papers compared with the rest of the databases which returned 202 papers in total. After evaluating the papers and performing a trial inclusion/exclusion sift, it was found that papers of any use were encapsulated in the other databases. One of the core benefits of Google Scholar is that it retrieves most open access papers, but the open access papers for this evaluation are all found in MDPI (a database for open access journal publishers). Science Direct and Scopus were both included in this paper even though they are both from the same umbrella parent company Elsevier. They use two different databases, with Science Direct containing full text articles from journals and books primarily published by Elsevier but also includes some hosted societies. Scopus on the other hand, indexes metadata from abstracts and references from thousands of publishers, including Elsevier, but not exclusively. Web of Science was also selected due to its more global coverage. By utilising the Web of Science platform as opposed to their core collection, you can access journals from non-English speaking regions such as Korea, Russia, Latin America and China, with a plethora of extra data sets on top of the core collection.

Each of these databases though do differ slightly in their search methods, preferring certain phrases and Boolean search terms and truncations compared to one another. Science Direct for example, doesn’t accept wildcards/truncations in their searches, meaning that phrases such as “Vertical Farm*” must be searched thrice; once as “Vertical Farm”, again as “Vertical Farms” and then finally as “Vertical Farming”. Each search term was entered individually, in combination with the other search terms. Scopus and Web of Science function very similarly when searching for publications. Both contain an advanced search which allows for combinations of phrases, as seen in Figure 1. By combining these searches into one larger search, it limits the number of individual searches needed to be completed, as it returns the same amount of hits as if we entered every combination

individually. A near identical system is available in Web of Science and the searches were completed in the same manner.

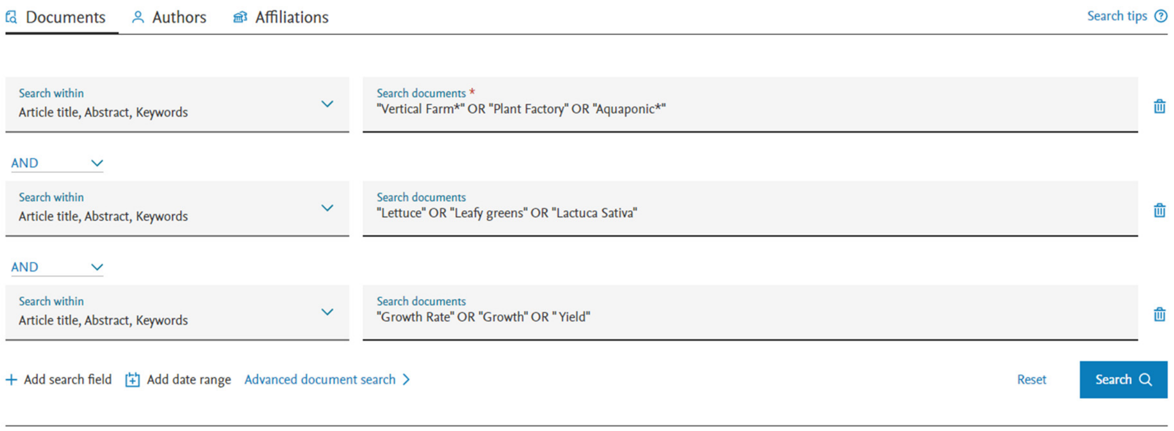

Figure S1: A screen capture of Scopus as an example of combining searches to limit the number of manual searches performed.

MDPI works more similarly to Science Direct, where individual searches will need to be carried out, as the advanced search function cannot combine searches. Unlike Science Direct though, it accepts truncations and wildcards in its search so multiple extra searches were not necessary.

The initial trial though did help identify other useful terms which could make my search more exhaustive. Within the search, I identified “Plant Factory” and “Salad greens”, both which have helped identify other papers not originally encapsulated. A full systematic search began on 12/02/2022, but when downloading papers from Science Direct, it was noted that only 1000 papers from each search could be downloaded and exported to be sifted. Because of this, slight methodological changes have occurred, now utilising four categories of search term as opposed to three which was utilised until the roadblock. Because of this, it allowed us to realize that the search string could be further refined to separate "Growing places” into “Growing methods” and “Growing place”. Though searches had already been performed for MDPI, WoS and Scopus, searches had to be performed again with slightly different search terms, to ensure a consistent methodological search.

All the publication databases have their differences in terms of search strategy and word preference, but all return searches of an equivalent value of if I had searched each term individually, except the removal of duplications already. Whilst knowing that all search engines equate to searching each search string individually, I performed 2880 individual searches, 720 per search database.

These searches resulted in 3706 individual references once duplicates were removed. These papers were then processed manually by whether they “fit” or “didn’t fit” the predefined inclusion/exclusion criteria, which will be stated below.

‘Let-us investigate; A meta-analysis of influencing factors on lettuce crop yields within CEA systems.’
Michael Gargaro, Richard J. Murphy, Zoe M. Harris

Rules for the sifting

The sifting of papers initially was done in two stages: by title, then abstract and full paper.

Initial YES, NO, MAYBE

For the title sifting, it needed to include the search terms utilised in the correct context. Here i had to take a common sense approach. I initially sorted them into YES, NO and MAYBE. YES includes papers i believe i need to read in more depth, NO means i wont ever read them as the titles are very far afield from the growth rate of lettuce. MAYBE, are papers whose titles are ambiguous, where i will go into more depth- intitially i read their abstracts before deciding if i need to read them in their entirety or not. Papers which are maybes were looked into first before the YES pile- so as to add them to the YES pile eventually

INCLUSION criteria for initial sift for complete reading

- Newer than 31st december 2008. (2009 onwards)

In abstract for sift of MAYBE, above plus :

- *Contain terms for ’growth’ related to ’lettuce (lactuca sativa)’. Inclusion even if ’growth rate’ or ’yield’ for example are not included explicitly, even if it just mentions ’growing lettuce’ or if ’lettuce grown in’ etc.*

EXCLUSION criteria for initial sift for complete reading

- Papers which explicitly are not looking at lettuce. Baby leaf/leafy greens/microgreens are included, otherwise discarded.
- Broken files, insufficient data in the citation.
- If in a different language to English and is not possible to accurately translate
- If not from a scientific, peer reviewed journal.

In abstract for sift of MAYBE, above plus:

- *If it contains a plant that isnt ’lettuce (lactuca sativa)’.*

From the initial YES, NO, MAYBE sift, there were 243 YES papers, 301 MAYBE papers and 3,019 NO papers. After the sifting of the MAYBE pile, i was left with 465 YES papers (which will be read through, and data extracted from.

At that time, there are:

0 MAYBE
465 YES
3,241 NO

Papers now ready to be fully read through, and data extracted.

Sifting of the YES pile (after Maybes eliminated into YES or NO) for inclusion in the Meta-analysis

INCLUSION criteria for meta-analysis
Contains primary data for extraction and utilisation in the MA.
Will need to contain data needed to calculate effect sizes.
Plus ALL criteria mentioned above also in the Title and Abstract sift.
SOME REVIEW PAPERS INCLUDED- the review papers should be included if they look relevant, i can utilise the paper later to read and extract data utilised in those review papers, which may not have

been encapsulated by the search.

EXCLUSION criteria for meta-analysis
Insufficient information for calculating effect sizes.

Data needed for the meta-analysis (extracted from the YES pile)

1. Characteristics of the studies
2. Data needed to calculate effect sizes
3. Study quality or risk of bias characteristics.

From the final sifting, 231 papers were excluded, leaving 234 papers from which data was extracted. Of these papers, not every single one had all data necesary for completing the meta-analysis. A list of these papers were collated and authors emailed with a request for data. If data was provided, it was included in the study. One email i recieved though was regarding the paper entitled ’ Intraspecific Variability Largely Affects the Leaf Metabolomics Response to Isosmotic Macrocation Variations in Two Divergent Lettuce (Lactuca sativa L.) Varieties’ by Corrado et al. This paper was replaced by his partner El-Nakhels paper entitled ‘The bioactive profile of lettuce produced in a closed soilless system as configured by combinatorial effects of genotype and macrocation supply composition’ as they were from the same paper, except El-Nakhel focussed on the growth factors, and Corrados more on the metabolomics. These 234 papers were then closer inspected to extract the data in pre-defined units for the meta-analysis, performing standard unit conversions if required. The data extraction parameters were chosen to allow the meta-analysis to be conducted (Table 1 in main text), but also to give further context to the meta-analysis results. In total, 121 papers were used in the final meta-analysis.

Appendix E

Germination times

Many of the papers within this study did not include germination times when reporting trial length, often referring to germination periods as reaching a certain number of leaves. To include these trials, educated assumptions of germination times were made so as to represent the full growing time in each trial. Assumptions for germination time were taken from other papers within this meta-analysis and in wider literature,

For every germination period stated, time period for the one leaf stage was 5 days, increasing by 5 days for every additional leaf (two leaf is 10 days, three leaf is 15 days etc). Though this is not perfect for every plant, every trial, or every cultivar, it is an informed guideline to use and is sufficient for this modelling, especially as the global analysis does not include a time factor.

Appendix F

Models used

Effect size calculations

To complete effect size calculations, a mean, sample size and standard deviation are necessary.

The arithmetic mean $\bar{x}$ is calculated by summing all individual values x_i in a sample and then dividing the sum by the sample size *n*.

$$\bar{x} = \frac{\sum_{i=1}^{n} x_i}{n} \quad (1)$$

For meta-analyses, we also need to calculate the standard error of the mean. To do this, we divide the sample standard deviation *s* through the square root of the sample size.

$$SE_x = \frac{s}{\sqrt{n}} \quad (2)$$

Effect sizes were pooled in R using a Random effects model to calculate the overall mean of the study or sample evaluated.

Appendix G

Author list with author name in Italics, and their corresponding papers title and DOI below.

‘Let-us investigate; A meta-analysis of influencing factors on lettuce crop yields within CEA systems.’
Michael Gargaro, Richard J. Murphy, Zoe M. Harris

Total Authors: 101
Total Papers: 122
Total Studies: 979

**Author name, paper and doi**
da Silva Cuba Carvalho et al.
**Influence of the use of wastewater on nutrient absorption and production of lettuce grown in a hydroponic system**
10.1016/j.agwat.2018.03.028
Ahmed et al.
**Evaluation of Lettuce (Lactuca sativa L.) Production under Hydroponic System: Nutrient Solution Derived from Fish Waste vs. Inorganic Nutrient Solution**
10.3390/horticulturae7090292
Albornoz et al.
**Effect of different day and night nutrient solution concentrations on growth, photosynthesis, and leaf NO3- content of aeroponically grown lettuce**
10.4067/S0718-58392014000200017
Amitrano et al.
**Modulating Vapor Pressure Deficit in the Plant Micro-Environment May Enhance the Bioactive Value of Lettuce**
10.3390/horticulturae7020032
Anderson et al.
**Growth and Tissue Elemental Composition Response of Butterhead Lettuce (Lactuca sativa, cv. Flandria) to Hydroponic and Aquaponic Conditions**
10.3390/horticulturae3030043
Awad et al.
**Biochar, a potential hydroponic growth substrate, enhances the nutritional status and growth of leafy vegetables**
10.1016/j.jclepro.2017.04.070
Baumbauer et al.
**Leaf lettuce yield is more sensitive to low daily light integral than kale and spinach**
10.21273/HORTSCI14288-19
Calone et al.
**Improving water management in European catfish recirculating aquaculture systems through catfish-lettuce aquaponics**
10.1016/j.scitotenv.2019.06.167
Carillo et al.
**Physiological and Nutraceutical Quality of Green and Red Pigmented Lettuce in Response to Cl Concentration in Two Successive Harvests**
10.3390/agronomy10091358
**Regulated salinity eustress in a Floating hydroponic module of sequentially harvested lettuce modulates phytochemical constitution, plant resilience, and post-harvest nutraceutical quality**
10.3390/agronomy11061040
Carotti et al.
**Plant Factories Are Heating Up: Hunting for the Best Combination of Light Intensity, Air Temperature and Root-Zone Temperature in Lettuce Production**
10.3389/fpls.2020.592171

**Pulsed led light: Exploring the balance between energy use and nutraceutical properties in indoor-grown lettuce**
10.3390/agronomy11061106
Chang et al.
**The growth response of leaf lettuce at different stages to multiple wavelength-band light-emitting diode lighting**
10.1016/j.scienta.2014.09.013
Cho et al.
**Growth and Bioactive Compounds of Lettuce as Affected by Light Intensity and Photoperiod in a Plant Factory Using External Electrode Fluorescent Lamps**
10.7235/HORT.20200059
Ciriello et al.
**Nutrient Solution Deprivation as a Tool to Improve Hydroponics Sustainability: Yield, Physiological, and Qualitative Response of Lettuce**
10.3390/agronomy11081469
Cometti et al.
**Cooling and concentration of nutrient solution in hydroponic lettuce crop**
10.1590/S0102-05362013000200018
Conversa et al.
**Reduction of Nitrate Content in Baby-Leaf Lettuce and Cichorium endivia Through the Soilless Cultivation System, Electrical Conductivity and Management of Nutrient Solution**
10.3389/fpls.2021.645671
**Soilless cultivation system, electrical conductivity of nutrient solution, and growing season on yield and quality of baby-leaf oak-leaf lettuce**
10.3390/agronomy11061220
Corrado et al.
**Configuration by Osmotic Eustress Agents of the Morphometric Characteristics and the Polyphenolic Content of Differently Pigmented Baby Lettuce Varieties in Two Successive Harvests**
10.3390/horticulturae7090264
**Phytochemical Responses to Salt Stress in Red and Green Baby Leaf Lettuce (Lactuca sativa L.) Varieties Grown in a Floating Hydroponic Module**
10.3390/separations8100175
Cristofano et al.
**Foliar and Root Applications of Vegetal-Derived Protein Hydrolysates Differentially Enhance the Yield and Qualitative Attributes of Two Lettuce Cultivars Grown in Floating System**
10.3390/agronomy11061194
da Rocha et al.
**Lettuce production in aquaponic and biofloc systems with silver catfish . Rhamdia quelen**
10.20950/1678-2305.2017.64.73
Egbuikwem et al.
**Assessment of suspended growth biological process for treatment and reuse of mixed wastewater for irrigation of edible crops under hydroponic conditions**
10.1016/j.agwat.2020.106034
El-Nakhel et al.
**An Appraisal of Urine Derivatives Integrated in the Nitrogen and Phosphorus Inputs of a Lettuce Soilless Cultivation System**
10.3390/su13084218

**Cultivar-Specific Performance and Qualitative Descriptors for Butterhead Salanova Lettuce Produced in Closed Soilless Cultivation as a Candidate Salad Crop for Human Life Support in Space**
10.3390/life9030061
**The bioactive profile of lettuce produced in a closed soilless system as configured by combinatorial effects of genotype and macrocation supply composition**
10.1016/j.foodchem.2019.125713
Etae et al.
**Effects of artificial light sources on growth and phytochemicals content in green oak lettuce**
10.1590/s0102-053620200213
Ezziddine et al.
**Quality and Yield of Lettuce in an Open-Air Rooftop Hydroponic System**
10.3390/agronomy11122586
Fallovo et al.
**Nutrient solution concentration and growing season affect yield and quality of Lactuca sativa L. var. acephala in Floating raft culture**
10.1002/jsfa.3641
Filho et al.
**Production of lettuce genotypes in hydroponic system using different organo-mineral nutrient solutions**
10.21475/ajcs.18.12.03.pne761
Frasetya et al.
**Application of rice husk silicate extract to increment growth of indoor hydroponic lettuce**
10.1088/1755-1315/542/1/012025
Gent et al.
**Composition of hydroponic lettuce: effect of time of day, plant size, and season**
10.1002/jsfa.4604
**Rate of change of composition of lettuce in response to nitrogen depletion or re-supply**
10.1002/jsfa.5716
Gimenez et al.
**An agroindustrial compost as alterative to peat for production of baby leaf red lettuce in a Floating system**
10.1016/j.scienta.2018.11.080
**Application of Directly Brewed Compost Extract Improves Yield and Quality in Baby Leaf Lettuce Grown Hydroponically**
10.3390/agronomy10030370
Giordano et al.
**Iron Biofortification of Red and Green Pigmented Lettuce in Closed Soilless Cultivation Impacts Crop Performance and Modulates Mineral and Bioactive Composition**
10.3390/agronomy9060290
Gizas et al.
**Impact of hydraulic characteristics of raw or composted posidonia residues, coir, and their mixtures with pumice on root aeration, water availability, and yield in a lettuce crop**
10.21273/hortsci.47.7.896
Goddek et al.
**Comparison of Lactuca sativa growth performance in conventional and RAS-based hydroponic systems**
10.1007/s10499-018-0293-8
Guilayn et al.

**Humic-like substances extracted from different digestates: First trials of lettuce biostimulation in hydroponic culture**
10.1016/j.wasman.2020.01.025
He et al.
**UV-A and FR irradiation improves growth and nutritional properties of lettuce grown in an artificial light plant factory**
10.1016/j.foodchem.2020.128727
Hernandez et al.
**Quality, Yield, and Biomass Efficacy of Several Hydroponic Lettuce (Lactuca sativa L.) Cultivars in Response to High Pressure Sodium Lights or Light Emitting Diodes for Greenhouse Supplemental Lighting**
10.3390/horticulturae6010007
Hikosaka et al.
**Dry-fog aeroponics affects the root growth of leaf lettuce (Lactuca sativa L. cv. Greenspan) by changing the flow rate of spray fertigation**
10.2525/ecb.53.181
Hooks et al.
**Effect of Pre-Harvest Supplemental UV-A/Blue and Red/Blue LED Lighting on Lettuce Growth and Nutritional Quality**
10.3390/horticulturae7040080
Hosseini et al.
**Nutrient Use in Vertical Farming: Optimal Electrical Conductivity of Nutrient Solution for Growth of Lettuce and Basil in Hydroponic Cultivation**
10.3390/horticulturae7090283
Huo et al.
**The influence of microalgae on vegetable production and nutrient removal in greenhouse hydroponics**
10.1016/j.jclepro.2019.118563
Jokinen et al.
**Root-applied glycinebetaine decreases nitrate accumulation and improves quality in hydroponically grown lettuce**
10.1016/j.foodchem.2021.130558
Joshi et al.
**A combination of downward lighting and supplemental upward lighting improves plant growth in a closed plant factory with artificial lighting**
10.21273/HORTSCI11822-17
Kanjamaneesathian et al.
**Spraying hydroponic lettuce roots with a suspension concentrate formulation of Bacillus velezensis to suppress root rot disease and promote plant growth**
10.30843/nzpp.2014.67.5734
Kappel et al.
**Ec sensitivity of hydroponically-grown lettuce (Lactuca sativa l.) types in terms of nitrate accumulation**
10.3390/agriculture11040315
Karnoutsos et al.
**Controlled root-zone temperature effect on baby leaf vegetables yield and quality in a Floating system under mild and extreme weather conditions**
10.1002/jsfa.11033
Kasozi et al.

**Effect of Bacillus spp. on Lettuce Growth and Root Associated Bacterial Community in a Small-Scale Aquaponics System**
10.3390/agronomy11050947
Kelly et al.
**Promotion of lettuce growth under an increasing daily light integral depends on the combination of the photosynthetic photon flux density and photoperiod**
10.1016/j.scienta.2020.109565
Khan et al.
**Exogenous Application of Amino Acids Improves the Growth and Yield of Lettuce by Enhancing Photosynthetic Assimilation and Nutrient Availability**
10.3390/agronomy9050266
Kleiber et al.
**Effect of ozone treatment and light colour on photosynthesis and yield of lettuce**
10.1016/j.scienta.2017.01.035
Kurashi et al.
**Growth control of leaf lettuce with exposure to underwater ultrasound and dissolved oxygen supersaturation**
10.1016/j.ultsonch.2018.10.005
La belle et al.
**Foliar Spray Application of Chlorella vulgaris Extract: Effect on the Growth of Lettuce Seedlings**
10.3390/agronomy11020308
Lau et al.
**Effects of Hydrogen Peroxide on Organically Fertilized Hydroponic Lettuce (Lactuca sativa L.)**
10.3390/horticulturae7050106
Leiber-Porcel et al.
**Elevated Root-Zone Dissolved Inorganic Carbon Alters Plant Nutrition of Lettuce and Pepper Grown Hydroponically and Aeroponically**
10.3390/agronomy10030403
Lennard et al.
**A Comparison of Plant Growth Rates between an NFT Hydroponic System and an NFT Aquaponic System**
10.3390/horticulturae5020027
Li et al.
**Effect of LED Spectrum on the Quality and Nitrogen Metabolism of Lettuce Under Recycled Hydroponics**
10.3389/fpls.2021.678197
Li et al.
**Growth and biomass yield of 25 crops in the 4-subject 180-day integrated experiment**
10.1016/j.actaastro.2019.06.028
Li et al.
**Growth Responses and Root Characteristics of Lettuce Grown in Aeroponics, Hydroponics, and Substrate Culture**
10.3390/horticulturae4040035
Li et al.
**Lettuce growth, nutritional quality, and energy use efficiency as affected by red-blue light combined with different monochromatic wavelengths**
10.21273/HORTSCI14671-19
Li et al.

**Optimization of rhizosphere cooling airflow for microclimate regulation and its effects on lettuce growth in plant factory**
10.1016/S2095-3119(20)63382-2
Li et al.
**Supplementary far-red and blue lights influence the biomass and phytochemical profiles of two lettuce cultivars in plant factory**
10.3390/molecules26237405
Liu et al.
**Growth and Nutrient Element Content of Hydroponic Lettuce are Modified by LED Continuous Lighting of Different Intensities and Spectral Qualities**
10.3390/agronomy10111678
**Regulation of accumulation and metabolism circadian rhythms of starch and sucrose in two leaf-color lettuces by red:blue ratios of LED continuous light**
10.1016/j.envexpbot.2022.104811***
Loconsole et al.
**Optimization of LED Lighting and Quality Evaluation of Romaine Lettuce Grown in An Innovative Indoor Cultivation System**
10.3390/su11030841
Martineau et al.
**Comparison of Light-emitting Diode and High-pressure Sodium Light Treatments for Hydroponics Growth of Boston Lettuce**
10.21273/HORTSCI.47.4.477
Maucieri et al.
**Effect of stocking density of fish on water quality and growth performance of European Carp and leafy vegetables in a low-tech aquaponic system**
10.1371/jourl.pone.0217561
Miceli et al.
**Effect of Gibberellic Acid on Growth, Yield, and Quality of Leaf Lettuce and Rocket Grown in a Floating System**
10.3390/agronomy9070382
Michelon et al.
**Strategies for Improved Yield and Water Use Efficiency of Lettuce (Lactuca sativa L.) through Simplified Soilless Cultivation under Semi-Arid Climate**
10.3390/agronomy10091379
Miller et al.
**Recycling Nutrient Solution Can Reduce Growth Due to Nutrient Deficiencies in Hydroponic Production**
10.3389/fpls.2020.607643
Modarelli et al.
**High Light Intensity from Blue-Red LEDs Enhance Photosynthetic Performance, Plant Growth, and Optical Properties of Red Lettuce in Controlled Environment**
10.3390/horticulturae8020114
Mohamed et al.
**The Impact of Light Spectrum and Intensity on the Growth, Physiology, and Antioxidant Activity of Lettuce (Lactuca sativa L.)**
10.3390/plants10102162
Monsees et al.

**Lettuce (Lactuca sativa, variety Salanova) production in decoupled aquaponic systems: Same yield and similar quality as in conventional hydroponic systems but drastically reduced greenhouse gas emissions by saving inorganic fertilizer**
10.1371/jourl.pone.0218368
Moraes et al.
**Behaviour of three lettuce cultivars in a hydroponic system**
10.15446/rfm.v73n2.75423
Nguyen et al.
**Effects of White LED Lighting with Specific Shorter Blue and/or Green Wavelength on the Growth and Quality of Two Lettuce Cultivars in a Vertical Farming System**
10.3390/agronomy11112111
Niu et al.
**Impact of Low and Moderate Salinity Water on Plant Performance of Leafy Vegetables in a Recirculating NFT System**
10.3390/horticulturae4010006
Noh et al.
**Optimizing temperature and photoperiod in a home cultivation system to program normal, delayed, and hastened growth and development modes for leafy oak-leaf and romaine lettuces**
10.3390/su131910879
Ohtake et al.
**Continuous irradiation with alternating red and blue light enhances plant growth while keeping nutritional quality in lettuce**
10.21273/HORTSCI13469-18
Orsini et al.
**Hydroponic lettuce yields are improved under salt stress by utilizing white plastic film and exogenous applications of proline**
10.1016/j.scienta.2018.01.019
Pannico et al.
**Combating Micronutrient Deficiency and Enhancing Food Functional Quality Through Selenium Fortification of Select Lettuce Genotypes Grown in a Closed Soilless System**
10.3389/fpls.2019.01495
Pennisi et al.
**Optimal photoperiod for indoor cultivation of leafy vegetables and herbs**
10.17660/eJHS.2020/85.5.4
**Resource use efficiency of indoor lettuce (Lactuca sativa L.) cultivation as affected by red:blue ratio provided by LED lighting**
10.1038/s41598-019-50783-z
Perio et al.
**Air Distribution in a Fully-Closed Higher Plant Growth Chamber Impacts Crop Performance of Hydroponically-Grown Lettuce**
10.3389/fpls.2020.00537
Pinho et al.
**Effluent from a biofloc technology (BFT) tilapia culture on the aquaponics production of different lettuce varieties**
10.1016/j.ecoleng.2017.03.009
Rouphael et al.

**Reducing Energy Requirements in Future Bioregenerative Life Support Systems (BLSSs): Performance and Bioactive Composition of Diverse Lettuce Genotypes Grown Under Optimal and Suboptimal Light Conditions**
10.3389/fpls.2019.01305
Sago et al.
**Quantitative Nutrient Management Reduces Nitrate Accumulation in Hydroponic Butterhead Lettuces Grown under Artificial Lighting**
10.21273/HORTSCI12418-17
**Zinc biofortification of hydroponic baby leaf lettuce grown under artificial lighting with elevated wind speed and root zone temperature**
10.2480/agrmet.D-17-00048
Saito et al.
**The effect of light quality on growth of lettuce**
10.3182/20101206-3-JP-3009.00052
Sakamoto et al.
**Effects of Salinity and Nutrients in Seawater on Hydroponic Culture of Red Leaf Lettuce**
10.2525/ecb.52.189
Samuoliene et al.
**The Physiological Response of Lettuce to Red and Blue Light Dynamics Over Different Photoperiods**
10.3389/fpls.2020.610174
Schwartz et al.
**Predictive Equations for Butterhead Lettuce (Lactuca Sativa, cv. Flandria) Root Surface Area Grown in Aquaponic Conditions**
10.3390/horticulturae5020039
Selma et al.
**Sensory quality, bioactive constituents and microbiological quality of green and red fresh-cut lettuces (Lactuca sativa L.) are influenced by soil and soilless agricultural production systems**
10.1016/j.postharvbio.2011.08.002
Semenova et al.
**The effect of plant growth compensation by adding silicon-containing fertilizer under light stress conditions**
10.3390/plants10071287
Shao et al.
**Alternation of temporally overlapped red and blue light under continuous irradiation affected yield, antioxidant capacity and nutritional quality of purple-leaf lettuce**
10.1016/j.scienta.2021.110864
Shimizu et al.
**Light Environment Optimization for Lettuce Growth in Plant Factory**
10.3182/20110828-6-IT-1002.02683
Shultz et al.
**Evaluation of different artificial light technologies for indoor aquaponic production of Bibb lettuce, Lactuca sativa var. capitata, and compact basil, Ocimum basilicum var. Genovese**
10.1111/jwas.12860
Söylemez et al.
**The impact of different growth media and ammonium-nitrate ratio on yield and nitrate accumulation in lettuce (Lactuca sativa var. longifolia)**
10.15835/nbha49412540
Spalholz et al.

**Impact of sun-simulated white light and varied blue:red spectrums on the growth, morphology, development, and phytochemical content of green- and red-leaf lettuce at different growth stages**
https://doi.org/10.1016/j.scienta.2020.109195
Suárez-Cáceres et al.
**Polyculture production of vegetables and red hybrid tilapia for self-consumption by means of micro-scale aquaponic systems**
10.1016/j.aquaeng.2021.102181
Sublett et al.
**Effects of Elevated Temperature and Potassium on Biomass and Quality of Dark Red ‘Lollo Rosso’ Lettuce**
10.3390/horticulturae4020011
**The Effect of Environment and Nutrients on Hydroponic Lettuce Yield, Quality, and Phytonutrients**
10.3390/horticulturae4040048
Sundar et al.
**Study on the Growth Performance of Lettuce (Lactuca sativa) and Pak Choi (Brassica chinensis) in Different Aquaponic Growing Systems**
10.3390/horticulturae6040069
Talukder et al.
**Application of alternating current electro-degradation improves retarded growth and quality in lettuce under autotoxicity in successive cultivation**
10.1016/j.scienta.2019.04.001
Thakulla et al.
**Nutrient Solution Temperature Affects Growth and °Brix Parameters of Seventeen Lettuce Cultivars Grown in an NFT Hydroponic System**
10.3390/horticulturae7090321
Tikasz et al.
**Aerated chicken, cow, and turkey manure extracts differentially affect lettuce and kale yield in hydroponics**
10.1007/s40093-019-0261-y
Touliatos et al.
**Vertical farming increases lettuce yield per unit area compared to conventional horizontal hydroponics**
10.1002/fes3.83***
Van Gerreway et al.
**Microbe-Plant Growing Media Interactions Modulate the Effectiveness of Bacterial Amendments on Lettuce Performance Inside a Plant Factory with Artificial Lighting**
10.3390/agronomy10101456
Voutsinos et al.
**Comparative Assessment of Hydroponic Lettuce Production Either under Artificial Lighting, or in a Mediterranean Greenhouse during Wintertime**
10.3390/agriculture11060503
Wang et al.
**Poultry biogas slurry can partially substitute for mineral fertilizers in hydroponic lettuce production**
10.1007/s11356-018-3538-1
**Wen et al.**
**Dynamic Responses of Ascorbate Pool and Metabolism in Lettuce to Light Intensity at Night Time under Continuous Light Provided by Red and Blue LEDs**
10.3390/plants10020214***
Wielgosz et al.

**Microbial Effects on the Production of Aquaponically Grown Lettuce**
10.3390/horticulturae3030046
Xin et al.
**Effects of environment lighting on the growth, photosynthesis, and quality of hydroponic lettuce in a plant factory**
10.25165/j.ijabe.20181102.3420
Yan et al.
**Evaluation of growth and quality of hydroponic lettuce at harvest as affected by the light intensity, photoperiod and light quality at seedling stage**
10.1016/j.scienta.2019.01.002
**Growth, nutritional quality, and energy use efficiency in two lettuce cultivars as influenced by white plus red versus red plus blue LEDs**
10.25165/j.ijabe.20201302.5135
**Growth, Nutritional Quality, and Energy Use Efficiency of Hydroponic Lettuce as Influenced by Daily Light Integrals Exposed to White versus White Plus Red Light-emitting Diodes**
10.21273/HORTSCI14236-19
Yang et al.
**Effects of ozone-treated domestic sludge on hydroponic lettuce growth and nutrition**
10.1016/S2095-3119(17)61868-9
Yang et al.
**Nutrient management regime affects water quality, crop growth, and nitrogen use efficiency of aquaponic systems**
10.1016/j.scienta.2019.108619
Zhang et al.
**Effect of divalent manganese (Mn2+) concentration on the growth and nitrate nitrogen content of lettuce during aeroponic intercropping with cherry radish**
10.1007/s13580-020-00303-0
Zhang et al.
**LED Light Quality of Continuous Light before Harvest Affects Growth and AsA Metabolism of Hydroponic Lettuce Grown under Increasing Doses of Nitrogen**
10.3390/plants10010176
Zhang et al.
**Supplemental Upward Lighting from Underneath to Obtain Higher Marketable Lettuce (Lactuca sativa) Leaf Fresh Weight by Retarding Senescence of Outer Leaves**
10.3389/fpls.2015.01110
Zhou et al.
**Photosynthetic characteristics and growth performance of lettuce (Lactuca sativa L.) under different light/dark cycles in mini plant factories**
10.32615/ps.2020.013

*** Additional papers to the total numbers written at the beginning of Appendix G. Used in VF calculations.
